# Kinetic evaluation of the solvolysis of isobutyl chloro- and chlorothioformate esters

**DOI:** 10.3762/bjoc.7.62

**Published:** 2011-04-29

**Authors:** Malcolm J D’Souza, Matthew J McAneny, Dennis N Kevill, Jin Burm Kyong, Song Hee Choi

**Affiliations:** 1 Department of Chemistry, Wesley College, 120 N. State Street, Dover, DE 19901-3875, USA; 2 Department of Chemistry and Biochemistry, Northern Illinois University, DeKalb, IL 60115-2862, USA; 3 Department of Chemistry & Applied Chemistry, Hanyang University, Ansan-si, Gyeonggi-do, 426-791, Korea

**Keywords:** addition–elimination, Grunwald–Winstein equations, ionization, isobutyl chloroformate, isobutyl chlorothioformate, solvolysis

## Abstract

The specific rates of solvolysis of isobutyl chloroformate (**1**) are reported at 40.0 °C and those for isobutyl chlorothioformate (**2**) are reported at 25.0 °C, in a variety of pure and binary aqueous organic mixtures with wide ranging nucleophilicity and ionizing power. For **1**, we also report the first-order rate constants determined at different temperatures in pure ethanol (EtOH), methanol (MeOH), 80% EtOH, and in both 97% and 70% 2,2,2-trifluoroethanol (TFE). The enthalpy (ΔH^≠^) and entropy (ΔS^≠^) of activation values obtained from Arrhenius plots for **1** in these five solvents are reported. The specific rates of solvolysis were analyzed using the extended Grunwald–Winstein equation. Results obtained from correlation analysis using this linear free energy relationship (LFER) reinforce our previous suggestion that side-by-side addition–elimination and ionization mechanisms operate, and the relative importance is dependent on the type of chloro- or chlorothioformate substrate and the solvent.

## Introduction

Alkyl chloro- and chlorothioformate esters are frequently used precursors [1–4] in the synthesis of pharmaceutical intermediates. Hence, it is important to comprehend the correlations between their chemical structure, chemical reactivity, and solvent effects. This knowledge can then be applied to the development of compounds that are designed to either stimulate or block other chemicals from interacting with targeted receptors. The effects of solvent variation upon the available specific rates of solvolysis of adamantyl [5–6], methyl [7], ethyl [8], 2,2,2-trichloro-1,1-dimethylethyl [9], *n*-propyl [10], isopropyl [11–12], *n*-octyl [13], and neopentyl [14] chloroformate esters, and those of methyl [15], ethyl [8], and isopropyl [16] chlorothioformate esters have been successfully analyzed using the extended [17–19] Grunwald–Winstein equation (Equation 1). In Equation 1, *k* and *k_0_* are the specific rates of solvolysis in a given solvent and in the standard solvent (80% ethanol), respectively, *l* estimates the sensitivity to changes in solvent nucleophilicity (*N*_T_), *m* represents the sensitivity to changes in the solvent ionizing power *Y*_Cl_, and *c* is a constant (residual) term.

[1]
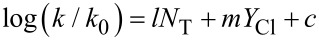


Kevill and Anderson developed *N*_T_ scales based on the solvolyses of the *S*-methyldibenzothiophenium ion [20–21] for considerations of solvent nucleophilicity, and Bentley et al. have recommended *Y*_Cl_ scales [22–25] based on the solvolyses of adamantyl derivatives for estimating the sensitivity to solvent ionizing power.

In reactions where the reaction center is adjacent to a π-system, or in α-haloalkyl aryl compounds that proceed via anchimeric assistance (*k*_∆_), Kevill and D’Souza proposed the addition of an aromatic ring parameter (*hI*) term [26–28] to Equation 1 to give Equation 2. In Equation 2, *h* represents the sensitivity of solvolyses to changes in the aromatic ring parameter *I*.

[2]
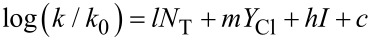


**Figure 1:**
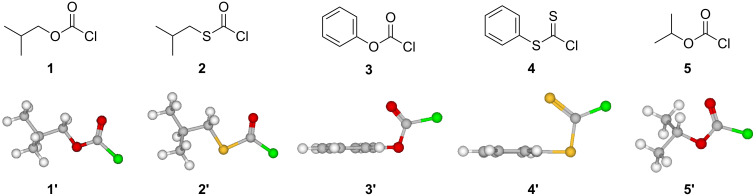
Molecular structures of *syn*-isobutyl chloroformate (**1**), *syn*-isobutyl chlorothioformate (**2**), phenyl chloroformate (**3**), phenyl chlorodithioformate (**4**), and isopropyl chloroformate (**5**). The 3-D images for *syn*-isobutyl chloroformate (**1'**), *syn*-isobutyl chlorothioformate (**2'**), phenyl chloroformate (**3'**), phenyl chlorodithioformate (**4'**), and isopropyl chloroformate (**5'**) are also shown.

Lee [29], Bentley [30] and others [3,31–38], used computational and experimental evidence to show that the chloroformate and chlorothioformate esters always exist in a *syn* conformation where the halogen atom is in a *trans* position with respect to the alkyl group. In Figure 1, the molecular structures for *syn*-isobutyl chloroformate (**1**), *syn*-isobutyl chlorothioformate (**2**), phenyl chloroformate (**3**), phenyl chlorodithioformate (**4**), and isopropyl chloroformate (**5**), and their corresponding 3-D structures **1'**, **2'**, **3'**, **4'** and **5'** are shown in the most stable geometries for RXCXCl (where X = S or O) which exist in a conformation where the C=X is *syn* with respect to R.

In a recent review [17], commemorating the 60^th^ anniversary of the Grunwald–Winstein equation, we previously published reported analyses [5–8,10–11,13,39–49] that were obtained using Equation 1, with examples of several alkyl and aryl chloro-, chlorothio-, chlorothiono-, and dithiochloroformate esters. For these esters, we proposed [17] side-by-side addition–elimination (A_N_ + D_N_) and ionization (S_N_1) solvolytic mechanisms, with proportions that were dependent on the type of RXCXCl (X = O or S) substrate, solvent nucleophilicity, and the ionizing ability of the solvents studied.

**Scheme 1:**
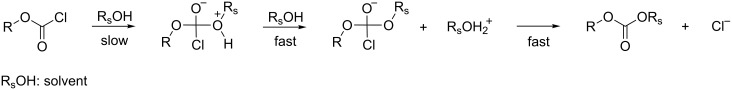
Stepwise addition–elimination mechanism through a tetrahedral intermediate for solvolysis of chloroformate esters.

**Scheme 2:**
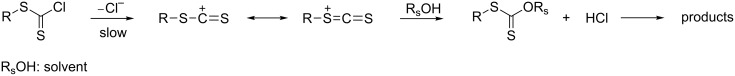
Unimolecular solvolytic pathway for the dithioformate esters.

At one extreme when R = Ph in phenyl chloroformate (PhOCOCl, **3**), due to the presence of two electronegative oxygen atoms and the planarity of the phenoxy group (**3'**), compound **3** [17,39–40] was found to solvolyze in all of the 49 solvents studied solely by an addition–elimination (A_N_ + D_N_) pathway (Scheme 1) with formation of the tetrahedral intermediate as the rate-determining step. When both oxygens are replaced by the more polarizable sulfur as in phenyl chlorodithioformate (PhSCSCl, **4**) [17,40,45], the mechanism of reaction was found to completely switch over to an ionization (S_N_1) pathway (Scheme 2) in all of the pure and binary aqueous organic mixtures studied. This tendency to follow an ionization process in such sulfur-for-oxygen substitutions occurs primarily as a result of the formation of a more favored resonance-stabilized transition-state (Scheme 2) [40,45].

We have since recommended [17] that the *l* (1.66) and *m* (0.56) values obtained by using Equation 1 for the solvolyses of **3**, and values of *l* (0.69) and *m* (0.95) obtained for the solvolyses of **4**, be taken as appropriate standards for the bimolecular addition–elimination and unimolecular ionization (without fragmentation) pathways, respectively. The appreciable sensitivity to solvent nucleophilicity (0.69) seen in the ionization–solvolysis of **4**, points to strong rear-side nucleophilic solvation of the developing resonance-stabilized carbocation. Another useful tool for mechanistic studies is the *l*/*m* ratio. We have found [17] that values >2.7 are typical of solvolytic mechanisms proceeding by an addition–elimination pathway with the addition-step being rate-determining (Scheme 1). Ratios between 0.5 and 1.0 signify a unimolecular ionization mechanism with strong rear-side nucleophilic solvation of the developing resonance-stabilized transition-state, while *l*/*m* values <<0.5 are indicative of an ionization–fragmentation process.

Early studies by other groups favored competing S_N_1 and S_N_2 pathways for the alkyl chloro-, chlorothio-, chlorothiono-, and dithiochloroformates [50–59]. Upon evaluating the rates of hydrolysis in aqueous solvents, Queen [54–55] suggested that with increasing electron donation to the chlorocarbonyl group in alkyl chloro- and chlorothioformates, the positive entropies and low solvent isotope effects pointed to a mechanism involving a unimolecular acyl–halogen bond fission. More recent studies on alkyl and aryl chlorothio-, chlorodithio-, and chlorothionoformate esters favor a stepwise mechanism via a zwitterionic tetrahedral intermediate [60–65].

Isobutyl chloroformate (**1**) and isobutyl chlorothioformate (**2**) have found use as specific precursors in novel synthetic routes for the preparation of peptidyl carbamate and thiocarbamate inhibitors of the enzyme elastase [66]. In Figure 1, the 3-D images of isobutyl chloroformate (**1'**) and isobutyl chlorothioformate (**2'**) are presented. In these figures, it is clear that the isopropyl group is pushed out of the plane due the presence of a carbon atom next to the ether or thioether atom in **1'** and **2'**. This could have an impact on any potential steric or electronic effects, due to presence of the isobutyl group, on the specific rates of reaction.

In this article we present determinations of the specific rates of reaction for isobutyl chloroformate (iBuOCOCl, **1**) at 40.0 °C and of isobutyl chlorothioformate (iBuSCOCl, **2**) at 25.0 °C in a variety of pure and binary aqueous organic solvents with wide ranging nucleophilicity and ionizing power values. Using Equation 1, we analyze in detail values for *l* and *m* obtained for **1** and **2** compared to those of the recommended standards (**3** and **4**) for such substrates, and also in comparison to the *l* and *m* values of other previously reported alkyl chloro- and chlorothioformate esters. We will also seek evidence for any changes in mechanism due to the presence of the isobutyl group. For **1**, we report studies at additional temperatures in five organic solvents to determine the corresponding values of the enthalpy (ΔH^≠^) and entropy (ΔS^≠^) of activation.

## Results and Discussion

The specific rates of solvolysis of **1** at 40.0 °C and of **2** at 25.0 °C, are reported in Table 1. Also presented in Table 1 are the *N*_T_ and *Y*_Cl_ values needed for the multiple correlation analysis of the assembled data using Equation 1.

**Table 1:** Specific rates of solvolysis (*k*) of isobutyl chloroformate (**1**) and isobutyl chlorothioformate (**2**), in several binary solvents and literature values for *N*_T_ and *Y*_Cl_.

Solvent^a^	**1** at 40.0 °C 10^4 ^*k* (s^−1^)^b^	**2** at 25.0 °C 10^5 ^*k* (s^−1^)^b^	*N*_T_^c^	*Y*_Cl_^d^

100% MeOH	3.28 ± 0.04	2.27 ± 0.14	0.17	−1.2
90% MeOH	6.25 ± 0.03	4.63 ± 0.22	−0.01	−0.20
80% MeOH	8.74 ± 0.08	7.57 ± 0.19	−0.06	0.67
70% MeOH	11.6 ± 0.2		−0.40	1.46
100% EtOH	0.848 ± 0.053	1.01 ± 0.09	0.37	−2.50
90% EtOH	1.97 ± 0.05	1.22 ± 0.10	0.16	−0.90
80% EtOH	2.65 ± 0.02	2.99 ± 0.13	0.00	0.00
70% EtOH	3.28 ± 0.02		−0.20	0.78
60% EtOH	4.19 ± 0.05		−0.38	1.38
50% EtOH	5.12 ± 0.05		−0.58	2.02
90% Acetone	0.113 ± 0.027		−0.35	−2.39
80% Acetone	0.316 ± 0.002	0.201 ± 0.015	−0.37	−0.80
70% Acetone	0.652 ± 0.004	1.06 ± 0.09	−0.42	0.17
60% Acetone	1.02 ± 0.02		−0.52	1.00
97% TFE (w/w)	0.0511 ± 0.0007	6.01 ± 0.10	−3.30	2.83
90% TFE (w/w)	0.0690 ± 0.0004	11.7 ± 0.8	−2.55	2.85
70% TFE (w/w)	0.263 ± 0.005	40.8 ± 2.3	−1.98	2.96
50% TFE (w/w)	0.775 ± 0.002		−1.73	3.16
80% T-20% E	0.0289 ± 0.0005	1.47 ± 0.09	−1.76	1.89
60% T-40% E	0.106 ± 0.001	0.688 ± 0.007	−0.94	0.63
50% T-50% E		0.299 ± 0.021	−0.64	0.60
40% T-60% E	0.283 ± 0.008	0.465 ± 0.016	−0.34	−0.48
20% T-80% E	0.561 ± 0.006	0.521 ± 0.027	0.08	−1.42
97% HFIP (w/w)		66.0 ± 2.9	−5.26	5.17
90% HFIP (w/w)		48.2 ± 1.6	−3.84	4.41
70% HFIP (w/w)		78.4 ± 2.0	−2.94	3.83

^a^Substrate concentration of ca*.* 0.0052 M; binary solvents on a volume–volume basis at 25.0 °C, except for TFE-H_2_O and HFIP-H_2_O (1,1,1,3,3,3-hexafluoro-2-propanol/water) solvents which are on a weight–weight basis. T-E are TFE-ethanol mixtures. ^b^With associated standard deviation. ^c^References [20–21]. ^d^References [22–25].

For **1**, we report in Table 2 the first-order rate constants determined at different temperatures in pure ethanol (EtOH), methanol (MeOH), 80% EtOH, 97% 2,2,2-trifluoroethanol (TFE) and 70% TFE. The corresponding enthalpy (ΔH^≠^) and entropy (ΔS^≠^) of activation values obtained from Arrhenius plots for **1** in these five mixtures are also reported in Table 2.

**Table 2:** Specific rates for solvolysis of isobutyl chloroformate (**1**) at various temperatures and the enthalpies and entropies of activation.

Solvent^a^	Temp. (°C)	10^4^ *k* (s^−1^)	ΔH^≠^ (kcal mol^−1^)^b^	ΔS^≠^ (cal mol^−1^K^−1^)^b^

100% MeOH	40.0	3.27 ± 0.05	14.1 ± 0.3	−29.6 ± 0.9
	45.0	4.63 ± 0.04		
	50.0	6.85 ± 0.06		
	55.0	9.54 ± 0.07		

100% EtOH	40.0	0.848 ± 0.005	15.2 ± 0.05	−28.6 ± 0.2
	45.0	1.27 ± 0.01		
	50.0	1.89 ± 0.02		
	55.0	2.71 ± 0.02		

80% EtOH	40.0	2.65 ± 0.02	14.0 ± 0.1	−30.4 ± 0.3
	45.0	3.85 ± 0.05		
	50.0	5.53 ± 0.05		
	55.0	7.732 ± 0.08		

70% TFE	40.0	0.263 ± 0.006	20.6 ± 0.4	−13.8 ± 1.3
	45.0	0.468 ± 0.004		
	50.0	0.775 ± 0.004		
	55.0	1.26 ± 0.01		

97% TFE	40.0	0.0511 ± 0.0007	21.5 ± 0.2	−14.3 ± 0.6
	55.0	0.266 ± 0.003		
	60.0	0.429 ± 0.009		
	65.0	0.704 ± 0.006		

^a^Volume–volume basis at 25.0 °C. ^b^With associated standard error.

The *l*, *m*, and *c* values obtained for **1** and **2**, together with the multiple correlation coefficients (*R*) and the *F*-test values are reported in Table 3, together with corresponding values from the literature for solvolyses of other chloroformate and chlorothioformate esters.

**Table 3:** Correlation of the specific rates of solvolysis of iBuOCOCl and iBuSCOCl (this study) and several other chloroformate and chlorothioformate esters (values from the literature), using the extended Grunwald–Winstein equation (Equation 1).

Substrate	*n*^a^	*l*^b^	*m*^b^	*c*^b^	*l*/*m*	*R*^c^	*F*^d^	Mechanism

PhOCOCl^g^	49	1.66 ± 0.05	0.56 ± 0.03	0.15 ± 0.07	2.95	0.980	568	A–E^e^
2-AdOCOCl^g^	19	0.03 ± 0.07	0.48 ± 0.04	−0.10 ± 0.09	0.06	0.971	130	I^f^
1-AdOCOCl^g^	11	0.08 ± 0.20	0.59 ± 0.05	0.06 ± 0.08	0.14	0.985	133	I^f^
MeOCOCl^g^	19	1.59 ± 0.09	0.58 ± 0.05	0.16 ± 0.07	2.74	0.977	171	A–E
EtOCOCl^g^	28	1.56 ± 0.09	0.55 ± 0.03	0.19 ± 0.24	2.84	0.967	179	A–E
	7	0.69 ± 0.13	0.82 ± 0.16	−2.40 ± 0.27	0.84	0.946	17	S_N_1
*n*-PrOCOCl^g^	22	1.57 ± 0.12	0.56 ± 0.06	0.15 ± 0.08	2.79	0.947	83	A–E
	6	0.40 ± 0.12	0.64 ± 0.13	−2.45 ± 0.27	0.63	0.942	11	S_N_1
iPrOCOCl^g^	9	1.35 ± 0.22	0.40 ± 0.05	0.18 ± 0.07	3.38	0.960	35	A–E
	16	0.28 ± 0.04	0.59 ± 0.04	−0.32 ± 0.06	0.47	0.982	176	I^f^
iBuOCOCl^h^	18	1.82 ± 0.15	0.53 ± 0.05	0.18 ± 0.07	3.43	0.957	82	A–E
neoPenOCOCl^g^	13	1.76 ± 0.14	0.48 ± 0.06	0.14 ± 0.08	3.67	0.977	226	A–E
	8	0.36 ± 0.10	0.81 ± 0.14	−2.79 ± 0.33	0.44	0.938	18	S_N_1
PhSCSCl^g^	31	0.69 ± 0.05	0.95 ± 0.03	0.18 ± 0.05	0.72	0.987	521	S_N_1
MeSCOCl^g^	12	1.48 ± 0.18	0.44 ± 0.06	0.08 ± 0.08	3.36	0.949	40	A–E
	8	0.79 ± 0.06	0.85 ± 0.07	−0.27 ± 0.18	0.93	0.987	95	S_N_1
EtSCOCl^g^	19	0.66 ± 0.08	0.93 ± 0.07	−0.16 ± 0.31	0.71	0.961	96	S_N_1
iPrSCOCl^g^	19	0.38 ± 0.11	0.72 ± 0.09	−0.28 ± 0.10	0.53	0.961	97	S_N_1
iBuSCOCl^i^	15	0.42 ± 0.13	0.73 ± 0.09	−0.37 ± 0.13	0.58	0.961	73	S_N_1
PhSCOCl^g^	16	1.74 ± 0.17	0.48 ± 0.07	0.19 ± 0.23	3.63	0.946	55	A–E
	6	0.62 ± 0.08	0.92 ± 0.11	−2.29 ± 0.13	0.67	0.983	44	S_N_1

^a^*n* is the number of solvents. ^b^With associated standard error. ^c^Multiple Correlation Coefficient. ^d^*F*-test value. ^e^Addition–elimination. ^f^Ionization–fragmentation. ^g^See text for references giving the source of this data. ^h^No 97–50% TFE. ^i^No 100%, 90% EtOH and MeOH, no 20% T-80% E.

As can be seen in Table 1, the pseudo first-order rate constants for **1** and **2** gradually increase as the amount of water is increased in the binary aqueous–organic solvents. This observation holds true even in the highly ionizing fluoroalcohols and can be attributed to solute–solvent interactions in the transition-state where both nucleophilicity and ionizing power play an important role. The very negative entropies of activation observed for **1** in the aqueous alcohols are typical for substrates that undergo solvolysis by a bimolecular process. The negative entropies of activation (−28.6 to −30.4 cal mol^−1^ K^−1^) in EtOH, MeOH and 80% EtOH are similar to those observed for the simplest primary alkyl chloroformate, methyl chloroformate (MeOCOCl) [67], where attack at the acyl carbon in an addition–elimination (A_N_ + D_N_) process was indicated as the rate-determining step. In order to evaluate the details of the interactions at the transition-state for **1**, we statistically analyzed (using Equation 1) the rates of reaction using multiple regression analysis. In all 22 solvents we obtained *l* = 1.11 ± 0.14, *m* = 0.43 ± 0.08, *R* = 0.886, *F*-test = 35, and *c* = 0.01 ± 0.10. The poor correlation coefficient and rather low *F*-test value was a strong indication of the possibility of superimposed dual mechanisms occurring within the range of solvent systems studied.

**Figure 2:**
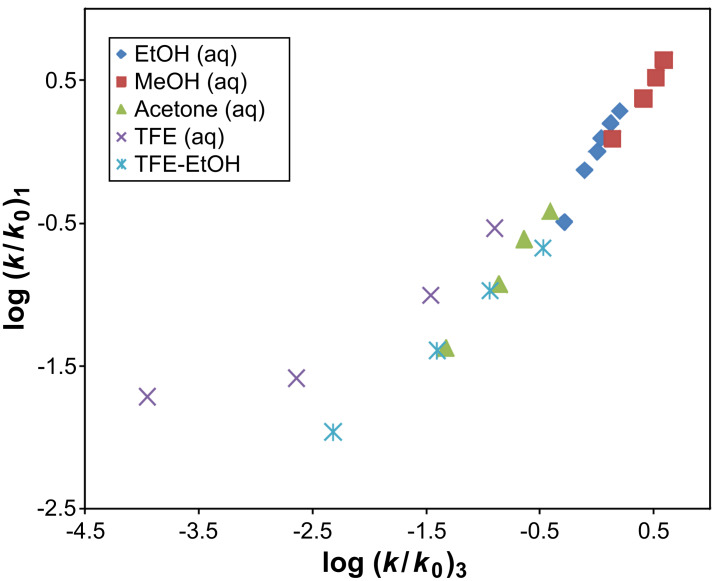
The plot of log (*k*/*k_0_*) for iBuOCOCl (**1**) against log (*k*/*k_0_*) for PhOCOCl (**3**).

As mentioned in the introduction, PhOCOCl (**3**) was shown to solvolyze in all of the 49 solvents studied by the addition–elimination process with a rate-determining addition step [39–40]. Using the similarity model concept [68], a plot of log (*k*/*k_0_*) for iBuOCOCl (**1**) in the 22 solvents studied against log (*k*/*k_0_*) for PhOCOCl (**3**) is shown in Figure 2. This plot results in a weak correlation with *R* = 0.913, *F*-test = 101, slope = 0.62 ± 0.06, and *c* = −0.08 ± 0.08. It is further observed in Figure 2 that the four aqueous TFE mixtures clearly lie above the line of best fit. Removal of these four points significantly improves the correlation analyses between **1** and **3** with results of *R* = 0.988, *F*-test = 659, slope = 0.947 ± 0.04 and *c* = −0.02 ± 0.03. This indicates that the mechanism of reaction for **1** and **3** in the remaining 18 pure and binary solvents (no aqueous TFE solvents) are identical.

**Figure 3:**
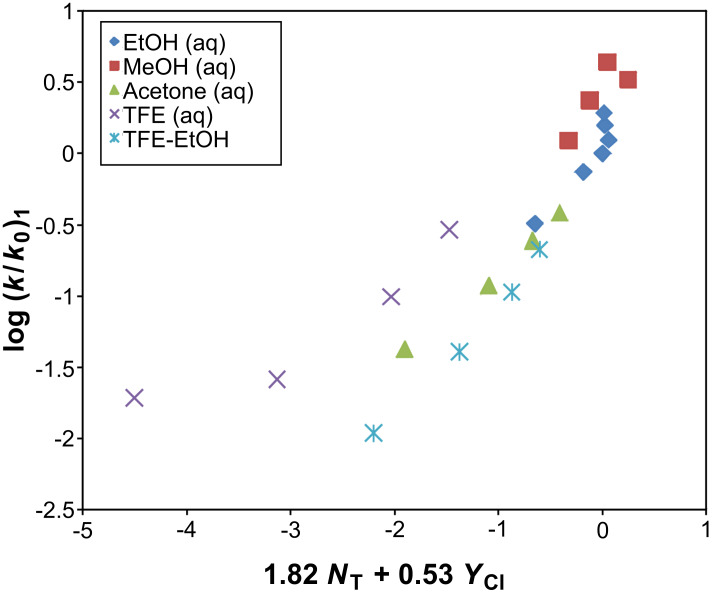
The plot of log (*k*/*k_0_*) for isobutyl chloroformate (**1**) against 1.82 *N*_T_ + 0.53 *Y*_Cl_ in eighteen pure and binary solvents. The points for the four aqueous TFE values were not included in the correlation. They are added here to show the extent of their deviation.

For **1**, analysis was performed for 18 solvents (no aqueous TFE) using Equation 1, and we obtained (reported in Table 3) *l* = 1.82 ± 0.15, *m* = 0.53 ± 0.05, *R* = 0.957, *F*-test = 82, and *c* = 0.18 ± 0.07. Such improvements seen in the correlation coefficient and *F*-test values for solvolyses of **1** on removal of the four aqueous TFE mixtures indicate that the data is now robust (Figure 3). The *l*/*m* ratio of 3.43 falls within the range (shown in Table 3) observed for the other alkyl chloroformate esters in the more nucleophilic solvents.

Previous solvolytic studies with primary alkyl chloroformates such as methyl chloroformate (MeOCOCl) [7], ethyl chloroformate (EtOCOCl) [8], and *n*-propyl chloroformate (*n*-PrOCOCl) [10] provided evidence that a bimolecular association–dissociation (addition–elimination) process was favored in the more nucleophilic solvents, while an ionization pathway was dominant in the highly ionizing solvents, including the fluoroalcohols with high fluoroalcohol content [7–8,10]. The *l*/*m* ratios for these three substrates in the more nucleophilic solvents (listed in Table 3) are almost identical in value (2.74, 2.84, and 2.79, respectively) and are very similar to the value of 2.94 observed for **3**.

The only two branched alkyl chloroformates that have been studied in detail using a Grunwald–Winstein analysis are isopropyl chloroformate (iPrOCOCl) [11–12] and neopentyl chloroformate (neoPenOCOCl) [14]. The secondary alkyl chloroformate, iPrOCOCl (**5**) [11–12], was found to solvolyze in a majority of the solvents studied by a mechanism similar to that proposed for the tertiary 1- or 2-adamantyl chloroformates [5–6]. This pathway included a unimolecular fragmentation–ionization process with loss of carbon dioxide [5–6,11–12]. For **5**, in nine of the more nucleophilic solvents the *l*/*m* ratio of 3.38 (Table 3) was a typical value for an addition–elimination (association–dissociation) mechanism [12].

We have proposed that neopentyl chloroformate (neoPenOCOCl) [14] solvolyzes in the HFIP rich mixtures with a Wagner–Meerwein 1,2-methyl shift leading to the formation of a tertiary pentyl cation. In 13 of the more nucleophilic solvents the *l*/*m* ratio of 3.67 (Table 3) for neoPenOCOCl was found to be typical of a bimolecular A_N_ + D_N_ process [14].

The higher errors associated with the *l* values, and the higher *l*/*m* ratios observed for iBuOCOCl (**1**), iPrOCOCl (**5**), and neoPenOCOCl, in the more nucleophilic solvents (3.43, 3.38, and 3.67, respectively) when compared to the *l*/*m* ratio obtained for **3** (2.95), is due to a limited range of solvents in which the A_N_ + D_N_ mechanism is operative. This view is supported by the multiple regression analysis of **3** in the same 18 solvents used for **1**, where an A_N_ + D_N_ mechanism is proposed, which yields *l* = 1.96 ± 0.14, *m* = 0.49 ± 0.05, *R* = 0.965, *F*-test = 101, and *c* = 0.23 ± 0.07 such that the *l*/*m* ratio for **3** is 4.00.

As shown in Table 1, the nucleophilicity (*N*_T_) values for the four aqueous TFE solvents range from a very low value of −3.30 in 97% TFE (w/w), to −1.73 in 50% TFE (w/w), while the ionizing power values (*Y*_Cl_) vary only slightly (2.83–3.16). A plot of log (*k*/*k_0_*)**_1_** against *N*_T_ in these four solvents results in a slope (*l*) = 0.72 ± 0.22 (0.08 probability that the term is statistically insignificant), *R* = 0.919, *F*-test = 11, and *c* = 0.51 ± 0.54. This *l* value is within the magnitude seen in aqueous fluoroalcohols for other alkyl chloroformate esters that undergo an ionization mechanism with strong rear-side solvation of the resonance-stabilized intermediate (Table 3).

In Table 4, the methanolysis and ethanolysis specifc rate order is shown to be *k*_MeOCOCl_ > *k*_EtOCOCl_ ≈ *k_n_*_-PrOCOCl_ ≈ *k*_iBuOCOCl_ ≈ *k*_OctOCOCl_ > *k*_iPrOCOCl_. As previously pointed out and shown in Figure 1, the presence of an additional carbon in the 3-D image of iBuOCOCl (**1'**), pushs the isopropyl group out of the plane of the ether oxygen. As a result, access to the carbonyl carbon in iBuOCOCl (**1'**) is not hindered by the presence of a branching alkyl group (**1'**, Figure 1), and the observed rate order in EtOH and MeOH (Table 4) suggests that any steric or inductive or hyperconjugative effect due to the presence of the isobutyl group in **1** is, at best, negligible. On the other hand, the inductive effect and competing hyperconjugative release of the isopropyl group in **5** does have an impact on its rates of ethanolysis and methanolysis.

**Table 4:** A comparison of the specific rates of solvolysis of MeOCOCl, EtOCOCl, *n*-PrOCOCl, iPrOCOCl, iBuOCOCl, and *n*-OctOCOCl in common solvents at 25.0 °C.

Solvent	MeOCOCl 10^5^ *k* (s^−1^)^a^	EtOCOCl 10^5^ *k* (s^−1^)^b^	*n*-PrOCOCl 10^5^ *k* (s^−1^)^c^	iPrOCOCl 10^5^ *k* (s^−1^)^d^	iBuOCOCl 10^5^ *k* (s^−1^)^e^	*n*-OctOCOCl 10^5^ *k* (s^−1^)^f^

100% MeOH	15.6	8.24	8.88	4.19	9.89	8.51
100% EtOH	3.51	2.26	2.20	1.09	2.36	2.39
80% EtOH	17.2	7.31	7.92	3.92	8.17	7.37
97% TFE		0.023	0.062	12.3	0.086	
70% TFE	0.857	0.611	0.591	19.7	0.481	

^a^Value obtained using Arrhenius plots with the values reported at different temperatures in reference [67]. ^b^Rates are reported at 24.2 °C in reference [8]. ^c^Reference [10]. ^d^Reference [12]. ^e^Extrapolated value obtained using Arrhenius plots with the values reported at different temperatures in Table 2. ^f^Reference [13].

**Figure 4:**
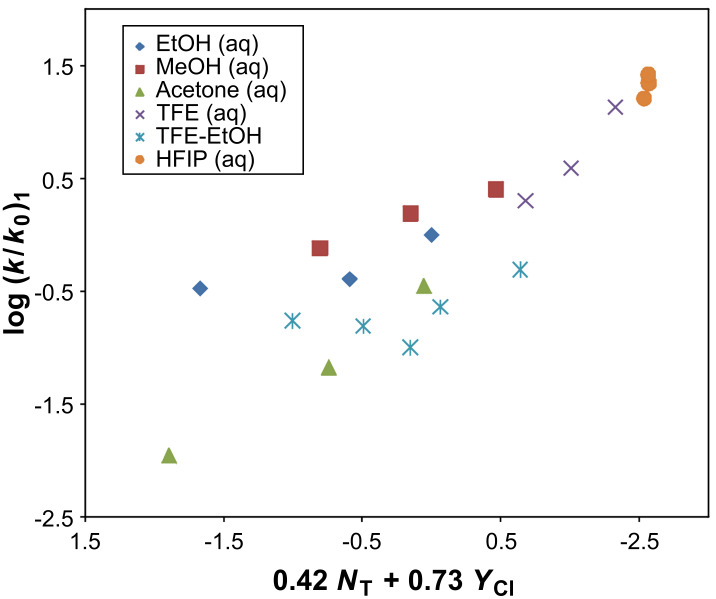
The plot of log (*k*/*k_0_*) for isobutyl chlorothioformate (**2**) against 0.42 *N*_T_ + 0.73 *Y*_Cl_ in 15 pure and binary solvents. The points for the 100% EtOH, 90% EtOH, 100% MeOH, 90% MeOH, and 20% T-80% E were not included in the correlation. They are added to show the extent of their deviation.

Grunwald–Winstein analysis using Equation 1 for isobutyl chlorothioformate (**2**) in all 20 solvents studied (Table 1) resulted in *l* = 0.34 ± 0.18, *m* = 0.57 ± 0.13, *R* = 0.873, *F*-test = 27, and *c* = −0.11 ± 0.17. This scatter can be resolved by excluding the rate data for **2** in 100% EtOH, 90% EtOH, 100% MeOH, 90% MeOH, and 20% T-80% E. In the remaining 15 solvents, the correlation coefficient (*R*) improves significantly to 0.961, the *F*-test value rises to 73, *l* = 0.42 ± 0.13, *m* = 0.73 ± 0.09, and *c* = −0.37 ± 0.13 (Table 3). A plot of log (*k*/*k_0_*) for isobutyl chlorothioformate (**2**) against 0.42 *N*_T_ + 0.73 *Y*_Cl_ is shown in Figure 4 with the five deviating points included.

The *l*/*m* ratio of 0.58 obtained for **2** in these 15 solvents is similar in magnitude to the previously observed ratios for methyl- (MeSCOCl) [15], ethyl- (EtSCOCl) [8], isopropyl- (iPrSCOCl) [16], and phenyl- (PhSCOCl) chlorothioformates [40,46–47] in solvents where an S_N_1 mechanism was said to be operative. The range of the *l*/*m* ratios, from 0.53 to 0.93, for these chlorothioformate esters (Table 3) is similar to the *l*/*m* ratio of 0.73 obtained for phenyl dithiochloroformate (**4**), the recommended standard for understanding ionization mechanisms in acyl containing systems.

Hence we suggest that in these 15 solvents, **2** solvolyzes by a dominant unimolecular ionization process with significant rear-side solvation of the developing acylium ion intermediate. For the five solvents (100% EtOH, 90% EtOH, 100% MeOH, 90% MeOH, and 20% T-80% E) whose data points lie above the regression line (Figure 4), a dominant superimposed addition–elimination mechanism (A_N_ + D_N_) is proposed. The rate order shown in Table 5, of *k*_MeSCOCl_ ≈ *k*_EtSCOCl_ ≈ *k*_iPrSCOCl_ ≈ *k*_iBuSCOCl_, is for the methanolysis and ethanolysis of these alkyl chlorothioformate esters at 25.0 °C. In pure methanol and ethanol a dominant association–dissociation (addition–elimination) mechanism, with rate-limiting addition, is believed to be effective in all four substrates. This rate order indicates that the inductive ability of the alkyl thioether group is almost independent of the type of alkyl group present. In Table 5, for solvolysis in the least nucleophilic and most highly ionizing solvent, 97% HFIP (w/w), a rate order of *k*_MeSCOCl_ << *k*_EtSCOCl_ < *k*_iBuSCOCl_ << *k*_iPrSCOCl_ is observed. This demonstrates that the hyperconjugative release during the formation of the developing resonance-stabilized carbocation intermediate is more efficient for isopropyl chlorothioformate (**5**) when compared to **2**, as the presence of the additional carbon pushes the isopropyl group out of the plane of the thioether atom in **2'** (Figure 1). This opinion is supported by an increase seen in the *l*/*m* ratio in the order of *k*_MeSCOCl_ < *k*_EtSCOCl_ < *k*_iBuSCOCl_ < *k*_iPrSCOCl_.

**Table 5:** A comparison of the rates of solvolysis of MeSCOCl, EtSCOCl, iPrSCOCl, and iBuSCOCl, in selected common solvents at 25.0 °C.

Solvent	MeSCOCl 10^5^* k* (s^−1^)^a^	EtSCOCl 10^5^ *k* (s^−1^)^b^	iPrSCOCl 10^5^ *k* (s^−1^)*^c^*	iBuSCOCl 10^5^ *k* (s^−1^)^d^

100% MeOH	2.00	2.15	1.99	2.27
100% EtOH	0.884	0.430	1.21	1.01
80% EtOH	2.44	2.68	13.7	2.99
97% TFE	0.986	5.98	49.8	6.01
90% TFE	1.92	10.2	69.5	11.7
70% TFE	13.9	54.3	212	40.8
97% HFIP	3.21	39.2	376	66.0
90% HFIP	3.48	36.1	437	48.2
70% HFIP	13.9	81.3	659	78.4

^a^Reference [15]. ^b^Rates are reported at 24.2 °C in reference [8]. ^c^Reference [16]. ^d^See Table 1.

As shown from Table 4 and Table 5, the *k*_iBuSCOCl_ < *k*_iBuOCOCl_ rate order applies in methanol and ethanol where the addition–elimination mechanism is dominant. This is due to the inductive ability of the isobutoxy group being much greater than that of the corresponding sulfur analog. The observed rate order is completely reversed in 97% TFE (aqueous) to *k*_iBuSCOCl_ >> *k*_iBuOCOCl_, where **2** is a 100-fold faster than **1**. In the highly ionizing fluoroalcohols an ionization mechanism (S_N_1) is proposed to prevail for both substrates: This rate order signifies that the hyperconjugative release from the sulfur atom in **2** to the developing acylium ion is the dominant factor.

## Conclusion

Correlation analysis of the solvolysis of isobutyl chloroformate (**1**) and isobutyl chlorothioformate (**2**) in a variety of pure and binary aqueous organic solvents was successfully analyzed using the extended Grunwald–Winstein equation (Equation 1). In both compounds side-by-side addition–elimination (with a rate-determining addition step) and unimolecular S_N_1 type mechanisms are believed to be possible.

In a majority of the solvents studied it is proposed that **1** solvolyzes by a bimolecular addition–elimination (A_N_ + D_N_) process due to the inductive ability of the isobutoxy group, whereas in the four aqueous TFE mixtures a predominant unimolecular S_N_1 mechanism with rear-side solvation of the developing carbocation is suggested.

For **2**, due to a more proficient hyperconjugative release, a dominant unimolecular ionization (S_N_1) mechanism with strong rear-side nucleophilic solvation is proposed for all solvents except 100% EtOH, 90% EtOH, 100% MeOH, 90% MeOH, and 20% T-80% E. In these five solvents an A_N_ + D_N_ process is believed to dominate.

## Experimental

The isobutyl chloroformate (98%, Sigma-Aldrich) and isobutyl chlorothioformate (96%, Sigma-Aldrich) were used as received. Solvents were purified and the kinetic runs carried out as previously described [5,39]. A substrate concentration of approximately 0.005 M in a variety of solvents was employed. The specific rates and associated standard deviations, as presented in Table 1, were obtained by averaging all of the values from duplicate runs.

Multiple regression analyses were carried out using the Excel 2007 package from the Microsoft Corporation. The 3-D-views presented in Figure 1, were generated using the KnowItAll^®^ Informatics System, ADME/Tox Edition, from BioRad Laboratories, Philadelphia, PA.
